# Fine-scale genetic structure and phenotypic divergence of a passerine bird population inhabiting a continuous Mediterranean woodland

**DOI:** 10.1098/rsos.240601

**Published:** 2024-06-12

**Authors:** Jorge Garrido-Bautista, Mar Comas, Michael J. Jowers, Steve Smith, Dustin J. Penn, Mohammed Bakkali, Gregorio Moreno-Rueda

**Affiliations:** ^1^ Department of Zoology, University of Granada, Granada 18071, Spain; ^2^ Department of Biological Sciences, Dartmouth College, Hanover, New Hampshire 03755, USA; ^3^ Department of Interdisciplinary Life Sciences, Konrad Lorenz Institute of Ethology, University of Veterinary Medicine Vienna, Vienna 1160, Austria; ^4^ Department of Genetics, Faculty of Sciences, University of Granada, Granada 18071, Spain

**Keywords:** blue tit, *Cyanistes caeruleus*, dispersal, genetic structure, neutral divergence, population differentiation

## Abstract

Genetic differentiation between populations inhabiting ecologically different habitats might appear because of limited dispersal and gene flow, which may lead to patterns of phenotypic divergence and local adaptation. In this study, we use dispersal, genotypic (24 microsatellite loci) and phenotypic (body size and clutch size) data to analyse patterns of genetic structuring and phenotypic divergence in a blue tit (*Cyanistes caeruleus*) population inhabiting a continuous and heterogeneous woodland along a valley. The two slopes of the valley differ in their forest formations and environmental conditions. Findings showed that most blue tits reproduced within their natal slope. Accordingly, microsatellite analyses revealed that populations of blue tits established in the two slopes show subtle genetic differentiation. The two genetic populations diverged in clutch size, exceeding the level of differentiation expected based on genetic drift, hence suggesting divergent selection (or other processes promoting divergence) on this life-history trait. Our findings reveal that restricted dispersal and spatial heterogeneity may lead to genetic differentiation among bird populations at a surprisingly small scale. In this respect, it is worth highlighting that such differentiation occurs for an organism with high dispersal capacity and within a continuous woodland. Moreover, we show that small-scale ecological differences, together with limited gene flow, can result in selection favouring different phenotypes even within the same continuum population.

## 1. Introduction

Dispersal is a key life-history trait playing a crucial role in determining the levels of gene flow among populations and thus adaptation to local environments [1]. Limited or restricted gene flow due to geographical barriers is regarded as the main cause of genetic divergence [2]. Indeed, genetic differentiation between populations typically increases with geographical distance [3–5]. Yet, genetic structuring can emerge at small scales because dispersal, and consequently gene flow, can be non-random [6] or because landscape configuration and local ecological conditions limit gene flow [7–9]. Limited gene flow is expected to enhance the capacity of populations to become adapted to local environmental conditions, whenever gene flow introduces foreign alleles that are less locally adapted [10,11].

Whenever individuals disperse from their natal territory, they must cope with novel environmental conditions to which they are not well adapted [12–14]. Accordingly, it is expected that selection against immigrant genes would reduce gene flow among populations, especially among contrasting environments, thus leading to genetic differentiation between populations, phenotypic divergence and local adaptation [14–19]. Population fragmentation because of restricted gene flow can favour the maintenance of local adaptations when genetic diversity is high enough in local populations [20,21]. Local adaptation could be constrained by immigrant genes from marginal and poor-quality habitats [22,23]. On the other hand, genetic differentiation may reduce the local genetic diversity and impact local adaptation if the effective population size is small [24]. In this way, dispersal can benefit populations by reducing close inbreeding as well as the consequent loss of genetic diversity [18,25,26].

Some studies show that non-random dispersal and genetic population differentiation can occur between neighbouring populations of highly mobile animals, such as birds [8,18,19,22,27–30]. Population genetic structuring has been shown to be accompanied by phenotypic differentiation in reproductive strategies, behaviour and morphology [14,15,17,31–35]. However, correlational studies are insufficient to make conclusions about patterns of local adaptation (36,37; but refer to [33]). There are logistic concerns whenever studying the adaptive significance of phenotypic divergence in birds (e.g. difficulty in carrying out common garden or reciprocal transplant experiments [38]). One way to study local adaptation is to measure both the neutral genetic divergence and the quantitative phenotypic variation among populations and compare them using the *P*_ST_–*F*_ST_ method, which allows one to infer the role of selection and genetic drift in shaping the phenotypic variation among populations [39–41]. The *P*_ST_–*F*_ST_ comparison quantifies the phenotypic differentiation (*P*_ST_) in relation to the level of divergence expected according to genetic drift alone (*F*_ST_). Previous studies using this approach found evidence of local adaptation in avian coloration, morphometry, behaviour and reproductive parameters [18,33,42–47]. Nonetheless, results derived from *P*_ST_–*F*_ST_ comparisons should be interpreted with caution, especially if the genetic markers used have high mutation rates [48].

In the present study, we analysed the natal dispersal, the genetic differentiation and the phenotypic divergence in a population of blue tits (*Cyanistes caeruleus*) inhabiting a continuous Mediterranean woodland located along a valley. The two slopes of the valley differ in forest formations and environmental conditions. Despite their potential for dispersal, blue tits only disperse over short distances, typically less than 1 km from their natal territory [18,30,49–51]. This restricted dispersion reduces gene flow between nearby blue tit populations and enhances genetic differentiation [8,19,30], which can lead to local adaptation [15,18,19]. Nonetheless, studies reporting a genetic structure among blue tit populations usually compared distant geographical populations or habitat patches in a mosaic landscape (1–28 km). The exceptions examining these processes in a continuous space in birds are the studies by Garant *et al*. [17] and Garroway *et al*. [52] that documented local adaptation and spatial genetic structure in the great tit (*Parus major*). Here, we show that limited dispersal can lead to genetic differentiation in a blue tit population inside a continuous woodland formed by different forest formations and without exhibiting any geographical barrier. Moreover, we found evidence of local adaptation in clutch size, since there was a divergence in this trait exceeding the level of divergence expected according to genetic drift alone.

## 2. Material and methods

### 2.1. Study area and sampling

**Figure 1.**
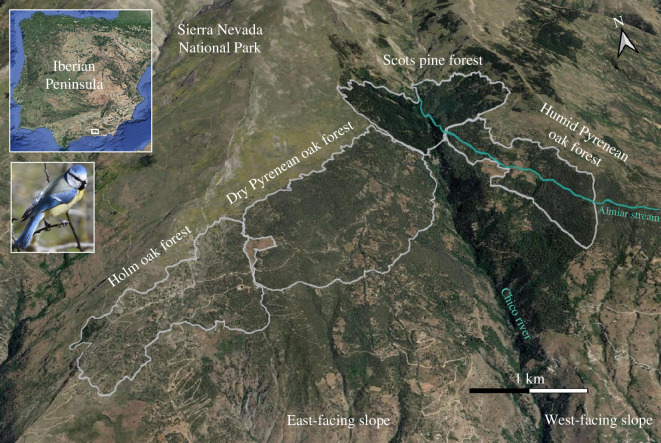
Map of the study area showing the valley where blue tits (*C. caeruleus*) reproduced. Note the difference in forest density and the presence of a stream crossing the west-facing slope. Map created using the software QGIS 3.10.5 connected to Google Earth.

Fieldwork was carried out during 2017, 2018 and 2019 in a woodland located at 1700–1800 m in the Sierra Nevada National Park (southeast Spain, 36°57′ N, 3°24′ W). This woodland is located along a valley. The east-facing slope of the valley is composed of two contiguous forest formations of Holm oaks (*Quercus ilex*) and Pyrenean oaks (*Q. pyrenaica*), while the west-facing slope is composed of Scots pines (*Pinus sylvestris*) and Pyrenean oaks (figure 1). Consequently, the two slopes differed in their environment (electronic supplementary material, appendix S1). The Pyrenean oak forests from the east-facing and west-facing slopes are henceforth referred to as dry and humid Pyrenean oak forests (figure 1).

Blue tits bred in nest boxes that were checked regularly to determine breeding parameters [53]. Birds were sexed and banded with aluminium rings for further identification. Birds were weighed to the nearest 0.1 g and their tarsus length was measured to the nearest 0.01 mm. We collected blood samples that were preserved in 1.5 ml tubes in absolute ethanol and stored at −20°C. In total, from 2017 to 2019, we placed 180 nest boxes on the east-facing slope (Holm oak forest: 40; dry Pyrenean oak forest: 140) and 210 nest boxes on the west-facing slope (Scots pine forest: 70; humid Pyrenean oak forest: 140). The occupation rate by blue tits was 62.5% in the Holm oak forest, 75.8% in the dry Pyrenean oak forest, 33.8% in the Scots pine forest and 74.4% in the humid Pyrenean oak forest. Capture–mark–recapture data from 2016 to 2019 were used to examine natal dispersal from their natal to their respective first-breeding nest box between the four forest formations and the two slopes of the valley. The number of ringed nestlings per nest box in each forest was: 214 in the Holm oak forest; 565 in the dry Pyrenean oak forest; 137 in the Scots pine forest; and 615 in the humid Pyrenean oak forest [53].

### 2.2. Microsatellite genotyping

We genotyped 171 blue tits (east-facing slope: 78, west-facing slope: 93). Ten microlitres of blood per blue tit was used for DNA extraction using the DNeasy Blood & Tissue kit (Qiagen, Hilden, Germany) following the manufacturer’s instructions except for the proteinase K digestion time, which was increased to 1 h, and the final elution volume, which was reduced to 50 µl. Twenty-seven polymorphic microsatellite markers were tested. Microsatellite genotyping is described in electronic supplementary material, appendix S2. According to microsatellite marker screening, three loci were discarded (electronic supplementary material, appendix S2), the remaining 24 loci being neutral to selection and used for subsequent genetic analyses.

Multi-locus genotypes were used to estimate the genetic diversity of the blue tit populations of the two slopes of the valley (i.e. the east-facing and the west-facing) and for the overall population. Estimates included the number of alleles (*K*), and the observed and expected heterozygosity (*H*_O_ and *H*_E_, respectively), calculated in ARLEQUIN 3.5 [54]. Genetic diversity estimates (*K*, *H*_O_, *H*_E_) were also calculated for all loci, including those that were not finally used in subsequent genetic analyses (electronic supplementary material, appendix S2).

We analysed patterns of spatial genetic structure using the model-based Bayesian Markov chain–Monte Carlo clustering method implemented in the software STRUCTURE 2.3.4 [55–57], which assigns individuals to inferred populations based on their multi-locus genotypes. We ran STRUCTURE assuming correlated allele frequencies, admixture and sampling locations (the two slopes of the valley) as priors. We conducted five independent runs for each value of cluster *K* (1–10), with 1 000 000 Markov chain–Monte Carlo (MCMC) iterations followed by a burn-in of 100 000 steps. The optimal number of genetic clusters was estimated using the log probabilities [Pr(*X*|*K*)] and Evanno’s Δ*K* method [58], implemented in STRUCTURE HARVESTER [59]. Optimal alignments of replicates of the same *K* and graphical representation of selected clusters were performed using the software CLUMPAK [60]. Since patterns of genetic structure and gene flow in the blue tit may be affected by male-biased philopatry [18,30], analyses of spatial genetic structure were performed both for all sampled individuals pooled together and considering male and female genotypes separately.

The inbreeding and outbreeding coefficients for each population were defined previously through the results of genetic population structuring conducted in STRUCTURE 2.3.4 [55–57]. The extent of geographical structuring of the genetic variation between individuals from the two slopes of the valley was evaluated based on *F*_ST_ statistics, using the analysis of molecular variance (AMOVA) [61], implemented in ARLEQUIN 3.5 [54]. The significance of variance components and *F*-statistics (*F*_ST_, *F*_IS_ and *F*_IT_) were assessed by 10 000 permutations. As above, *F*-statistics were also calculated considering male and female genotypes separately.

### 2.3. Phenotypic population differentiation

Previous research found that females adjusted their clutch size to the slope of the valley where they bred [53]. Here, we used the *F*_ST_–*Q*_ST_ (*P*_ST_) approach to examine the relative importance of genetic drift and natural selection in explaining the variation in such life-history (clutch size) and morphological (body mass and tarsus length) traits between the two slopes of the valley. *F*_ST_ estimates the extent of population genetic differentiation, while *Q*_ST_ estimates the differentiation of quantitative genetic traits [62]. However, *Q*_ST_ uses purely additive genetic variance, and its estimation requires rearing individuals from different populations in a common environment [33], which was unfeasible for this study. For this reason, we used *P*_ST_, analogous to *Q*_ST_, to quantify the between populations variance in quantitative phenotypic traits [39,63]. We calculated the phenotypic differentiation (*P*_ST_) for each quantitative trait: clutch size, body mass and tarsus length (electronic supplementary material, appendix S3).

The *F*_ST_–*P*_ST_ comparisons allow the detection of local adaptation by testing the relationship between neutral and quantitative genetic variation among populations. There are three possible outcomes from the *F*_ST_–*P*_ST_ comparisons [64,65]. If *P*_ST_ is higher than *F*_ST_, then the degree of divergence in phenotypic quantitative traits exceeds that achievable by genetic drift alone (measured through neutral markers), and consequently, natural selection favours different phenotypes in every different population (diversifying selection). If *P*_ST_ equals *F*_ST_, then the relative effects of natural selection and genetic drift are indistinguishable, hence natural selection is not implied in population differentiation. Lastly, if *P*_ST_ is lower than *F*_ST_, then natural selection is favouring the same phenotype in different populations, so phenotypic differentiation is less than expected on the basis of neutral divergence [65]; see electronic supplementary material, appendix S3, for more details.

## 3. Results

### 3.1. Natal dispersal

We recaptured 11 blue tits that were ringed as nestlings, seven individuals at the east-facing slope of the valley and four at the west-facing slope. Only one blue tit (1 out of 11; 9.09%) dispersed between slopes, being born in the west-facing slope (humid Pyrenean oak forest) and reproducing for the first time in the east-facing slope (dry Pyrenean oak forest).

### 3.2. Genetic population differentiation and structure

Between 2 and 40 alleles were detected per locus (mean = 14), with a mean *H*_O_ of 0.58 (range = 0.05–0.87) and a mean *H*_E_ of 0.73 (range = 0.45–0.95) (electronic supplementary material, table S1). The mean *H*_O_ of the populations of the east-facing and west-facing slopes were 0.58 and 0.54, respectively, and their mean *H*_E_ were 0.73 and 0.70, respectively.

The geographical structuring inferred from the microsatellite markers between the populations of the two slopes of the valley revealed that most of the variation lies within individuals (table 1). The fixation index *F*_ST_ among populations indicated a significant genetic divergence between both slopes (*F*_ST_ = 0.016, *p* < 0.001, 1.57% of the variance; table 1). In accordance, the *F*_IS_ estimate significantly differed from zero (average *F*-statistic over all loci, *F*_IS_ = 0.220, *p* < 0.001, 18.94% of the variance; table 1), suggesting outbreeding within both genetic populations. Also, *F*_IS_ estimates of each genetic population were statistically significantly different from zero (east-facing slope = 0.176, west-facing slope = 0.207; in both cases, *p* < 0.001). Lastly, the *F*_IT_ estimate indicated that most of the variation lies within individuals (average *F*-statistic over all loci, *F*_IT_ = 0.231, *p* < 0.001, 79.5% of the variance; table 1). When considering male and female genotypes separately, the same pattern emerged (*F*_ST_ for males = 0.018, *p* < 0.001; *F*_ST_ for females = 0.015, *p* < 0.001; *F*_IS_ for males = 0.203, *p* < 0.001; *F*_IS_ for females = 0.188, *p* < 0.001; *F*_IT_ for males = 0.218, *p* < 0.001; *F*_IT_ for females = 0.200, *p* < 0.001; table 1).

**Table 1.** Hierarchical AMOVAs for the two genetic blue tit (*C. caeruleus*) populations analysed by microsatellite marker data, considering all sampled individuals and male and female genotypes separately.

source of variation	degrees of freedom	sum of squares	variance components	percentage of variation
**all individuals**				
among populations	1	29.426	0.120	1.57
among individuals within populations	169	1522.840	1.454	18.94
within individuals	171	1043.500	6.102	79.49
total	341	2595.766	7.677	100
**male genotypes**				
among populations	1	20.814	0.148	1.88
among individuals within populations	76	705.257	1.566	19.92
within individuals	78	479.500	6.147	78.20
total	155	1205.571	7.862	100
**female genotypes**				
among populations	1	19.161	0.112	1.45
among individuals within populations	91	817.812	1.424	18.55
within individuals	93	571.00	6.139	80.00
total	185	1407.973	7.675	100

**Figure 2.**
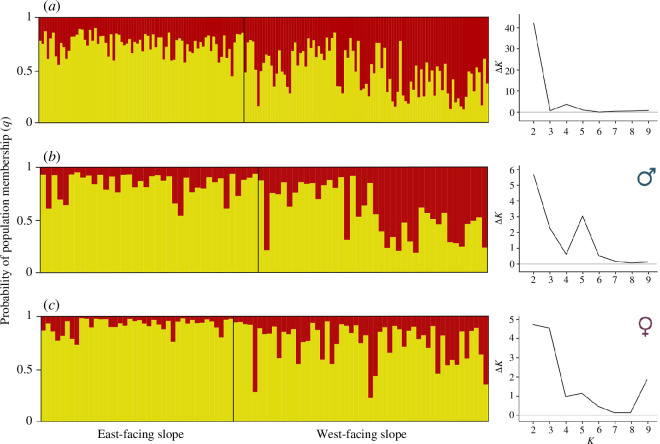
Genetic assignment based on the Bayesian clustering analysis implemented in STRUCTURE software, considering (*a*) all individuals, (*b*) males only and (*c*) females only. Each individual is represented by a vertical line, which is partitioned into two coloured segments each representing the individual’s probability of pertaining to one of the two populations (*q*) that the genetic clustering gave. Right: Bayesian clustering shows the most probable number of genetic clusters according to Δ*K* values.

Structure analyses considering all individuals revealed a maximum Δ*K* and Pr(*X*|*K*) for *K* = 2, indicating the presence of two genetic clusters across the woodland (figure 2*a*). Genetic differentiation occurred mainly between the populations of the east-facing and west-facing slopes of the valley (figure 2*a*). Analyses considering male and female genotypes separately also revealed a maximum Δ*K* and Pr(*X*|*K*) for *K* = 2 (figure 2*b,c*, respectively), with a similar pattern of genetic structure to that observed in the analysis considering all the sampled individuals. Overall, genetic population differentiation and structure analyses indicated the presence of subtle genetic differentiation within the woodland.

### 3.3. Phenotypic population differentiation

When considering the null assumption where *g*/*h* ^2^ = 1 (i.e. when the proportion of variance between populations due to additive genetic effects equals the proportion of variance due to genetic effects within populations), only the *P*_ST_ estimate for clutch size (0.052) was higher than the global pairwise *F*_ST_ (body mass: 0.013, tarsus length: 0.005). Hence, comparisons of quantitative trait differentiation (*P*_ST_) with its expectation under neutrality (*F*_ST_) revealed evidence of divergent selection for clutch size (*P*_ST_ > *F*_ST_). The *P*_ST_ values for clutch size were higher than the global *F*_ST_ when altering the assumptions about heritability and the values of additive genetic proportion. On the other hand, the *P*_*ST*_ values for tarsus length never exceeded the global *F*_ST_, while the *P*_ST_ values for body mass were higher than the global *F*_ST_ only at medium to high *g* values (*g* > 0.4) when heritability was relatively low (electronic supplementary material, appendix S3). Therefore, the results from *F*_ST_–*P*_ST_ comparisons suggest that clutch size was under diversifying selection.

## 4. Discussion

Our findings reveal that, despite having a high dispersal capacity, blue tits in our study sites showed genetic and phenotypic differentiation within a continuous population. This pattern was mainly driven by non-random dispersal—as suggested by both the genetic and the capture–mark–recapture data—associated with environmental heterogeneity within the woodland. Previous studies already showed that capture–mark–recapture data provide accurate information on recent levels of gene flow among bird populations [16,18,30,66–68]; but see [69]. Widespread highly mobile species are likely to experience several environmental conditions and geographical barriers, and thus, they are expected to experience genetic population structure at large geographical scales [70–73], often following an isolation-by-distance pattern (e.g. [3,72,74]). However, genetic structure can be observed at small spatial scales depending on the landscape composition and environmental conditions [8], which can restrict dispersal and gene flow among populations when organisms match habitat selection to natal conditions [75]. Hence, our results show that genetic structure (and probably local adaptation) can occur even among smaller population patches than previously thought (but see [17]), since we showed subtle genetic structuring associated with environmental heterogeneity even within the same woodland and for a species with a potential high dispersal capacity.

The blue tits inhabiting the continuous woodland displayed a limited dispersal, with all individuals, except for one, reproducing within their natal slope of the valley. The strong philopatry of blue tits can explain the genetic differentiation among populations on the two slopes of the valley covered by the woodland, although we acknowledge that the reduced recapture rate precludes us from examining accurate gene flow estimates and other dispersal dynamics. Blue tits often breed with related partners and females show less promiscuity with genetically distant males ([49]; see also [76]), contributing to the genetic population structure and potentially leading to local adaptation [77]. Our results were similar to other studies that examined patterns of genetic structure between neighbouring and fragmented populations of tits (Paridae) (table 2) and other passerine birds [27,32,88]. However, we found a level of genetic differentiation higher than expected given the extremely short distance that separates the two slopes of the valley (see table 2 for geographical and genetic distances in other blue tit populations), and the fact that the two slopes of the valley are connected through a pine forest formation and, thus, form a continuum (figure 1).

**Table 2.** Summary of studies reporting the geographical and the genetic distance among natural populations of tits (Paridae), namely, blue tits (*C. caeruleus*), great tits (*P. major*) and black-capped chickadees (*P. atricapillus*). The table summarizes the studies examining genetic structure among populations at medium to low geographical distances (continental large-scale studies are excluded).

species	geographical distance (km)^a^ ^,b^	genetic distance (*F*_ST_ values)^a^ ^,c^	habitat types	genetic marker	reference
blue tit (*C. caeruleus*)	0 km	**0.016**	continuous woodland	microsatellites	This study
	<2 km	0.006	mosaic of forest patches	microsatellites	Ferrer *et al*. [78]
	1–28 km	0.000–0.017	mosaic of forest patches	microsatellites	Ferrer *et al*. [8]
	2–90 km	0.000–**0.010**	mosaic of forest patches	minisatellites	Verheyen *et al*. [79]
	5–440 km	**0.008–0.087**	mosaic of forest patches	SNPs	Perrier *et al*. [73]
	5–440 km	0.009–**0.054**	mosaic of forest patches	SNPs	Szulkin *et al*. [80]
	5–493 km	0.001–0.049	mosaic of forest patches	microsatellites	Porlier *et al*. [19]
	5–9 km	**0.028–0.063^d^**	urban parks and forest patch	microsatellites	Senar & Björklund [81]
	7 km	**0.005**	mosaic of forest patches	microsatellites	Ferrer *et al*. [82]
	7 km	**0.033**	mosaic of forest patches	microsatellites	García-Navas *et al*. [18]
	10–50 km	0.002–0.004	urban parks and forest patches	microsatellites	Markowski *et al*. [83]
	20 km	**0.003**	mosaic of forest patches	microsatellites	Ortego *et al*. [30]
	25 km	−0.003 and **0.012^e^**	mosaic of forest patches	microsatellites	Blondel *et al*. [84]
	25 km	**0.004**	mosaic of forest patches	SNPs	Dubuc-Messier *et al*. [33]
	Not available	**0.007–0.021**	mosaic of forest patches	microsatellites	Blondel *et al*. [22]
great tit (*P. major*)	500 m–3 km	0.000–**0.190**	urban parks and forest patch	microsatellites	Björklund *et al*. [28]
	5–150 km	0.003–**0.011**	mosaic of forest patches	microsatellites	Postma *et al*. [85]
	7 km	0.006	mosaic of forest patches	microsatellites	García-Navas *et al*. [86]
	10–50 km	0.003–**0.010**	urban parks and forest patches	microsatellites	Markowski *et al*. [83]
	500 km	**0.010**	mosaic of forest patches	SNPs	van Bers *et al*. [87]
black-capped chickadee (*P. atricapillus*)	26–1500 km	**0.009**–0.316	not reported	microsatellites	Adams *et al*. [71]

^a^ When more than two populations were compared, the table shows a range of values (minimum to maximum geographical distance, and minimum to maximum *F*_ST_ values).

^b^ When geographical distances among populations were not reported in the original study, Euclidean distances were estimated based on coordinates and map information.

^c^ Statistically significant pairwise *F*_ST_ values are marked in bold.

^d^ The study reported *G*_ST_ values instead of *F*_ST_ values.

^e^ The study reported the *F*_ST_ values in two separate years.

Despite the statistically significant genetic differentiation of the two subpopulations, blue tits still showed some degree of outbreeding within both slopes of the valley, suggesting the existence of some gene flow, an expected finding if one considers the short geographical distance that separates the two blue tit subpopulations [30]. This scenario, *a priori*, could prevent genetic and phenotypic divergence as gene flow might introduce maladaptive alleles into the locally adapted populations [10,11]. Nonetheless, divergent selection can maintain local adaptation and adaptive divergence despite the homogenizing effects of gene flow [89–91], especially when environmental heterogeneity generates spatially contrasting selection pressures [6,43]. This would occur if local populations retain high genetic diversity [20,21]. The blue tits inhabiting the continuous woodland studied here exhibited moderate to high levels of genetic diversity (heterozygosity) both at population and loci levels (see §3 and electronic supplementary material, appendix S2), and gene flow seems to maintain such genetic diversity in each slope of the valley. Several studies have revealed significant associations between individual genetic quality and several fitness-related traits in blue tits, such as the probability of infection by parasites [92], carotenoid-based feather coloration [78,93], egg quality [93], clutch size [94] and the probability of local recruitment [82,94]. Therefore, it seems that reproductive success is not constrained by potential genetic inbreeding within each subpopulation, while the phenotypic divergence between both subpopulations still seems to be maintained by diversifying selection.

We found some evidence that blue tits were locally adapted to the conditions of the slope they inhabit, as clutch size was differentiated between both slopes of the valley to a greater extent than expected if we consider genetic drift alone (i.e. *P*_ST_ > *F*_ST_), suggesting that selection favours different clutch sizes in the two slopes of the valley. These findings are congruent with a previous study in which we found that females adjust their clutch size to the rearing conditions in the slope where they bred, thus maintaining the breeding success constant throughout the woodland [53]. Concretely, females from the east-facing slope lay on average one egg less than females from the humid Pyrenean oak forest on the west-facing slope [53]. The heritability of clutch size is high in the blue tit [18] and moderately high in other passerine species [95,96], indicating a substantial additive genetic variance for this life-history trait that, hence, provides considerable options and responses for selection to act on. However, studies reporting phenotypic differentiation in avian traits, including the clutch size, exceeding the level of genetic neutral divergence rely on large scales across the species breeding ranges ([42–47]; but see [18]). In fact, few studies have found evidence of local adaptation in bird populations at such small scales (table 2). Garant *et al*. [17] showed that non-random gene flow and divergent selection generate genetic differentiation in great tit nestling body mass in a continuous woodland. Similarly, Garroway *et al*. [52] found evidence of fine-scale genetic structure associated with malaria infection risk and local conspecific density. Postma and van Noordwijk [14] and Postma *et al*. [85,97] showed that non-random gene flow is the main cause for the genetic differences in the clutch size between two island great tit populations only 1.3 km apart. Regarding blue tits, García-Navas *et al*. [18] found that the divergence in clutch size and morphological traits exceeded the neutral genetic differentiation in two blue tit populations separated by 7 km. Charmantier *et al*. [98] and Blondel *et al*. [22] showed evidence of local adaptation for several traits occurring in blue tit populations inhabiting forest patches separated by a few kilometres. Finally, Camacho *et al*. [31] documented a genetic and phenotypic divergence for tarsus length at a short spatial distance (1.1 km) in two pied flycatcher (*Ficedula hypoleuca*) populations. Our findings and the aforementioned studies reveal that phenotypic divergence can occur over surprisingly small spatial scales even for birds, with high potential for dispersal. However, to our knowledge, only the present study and those by Garant *et al*. [17] and Garroway *et al*. [52] provide evidence for such differentiation in phenotypic traits (clutch size, body mass and resistance to *Plasmodium* parasites, respectively) over continuous systems. Still, the results derived from the *P*_ST_–*F*_ST_ comparison should be interpreted with caution since the analyses were run using microsatellites, genetic markers which have typically high mutation rates and hence can bias the *F*_ST_ estimate [48]. Other processes apart from divergent selection, such as habitat match selection to natal conditions [75], may operate to produce the observed clutch size differentiation. Additional experiments would be needed to test these hypotheses.

Environmental heterogeneity within the woodland, together with limited dispersal, seems to be behind the evolutionary process disentangled in this study. The study area’s topography results in a microscale geographic heterogeneity similar to the evolution canyon models described in [99,100]. In these systems, the opposite and closely neighbouring slopes of a valley display marked climatic and biotic contrasts which determine the ecological and evolutionary processes developing on each slope. The east-facing slope of the woodland studied here receives more solar radiation, has a higher temperature and lower humidity, less tree cover, lower nest infestation by nest-dwelling ectoparasites and a lower caterpillar abundance during the spring compared with the west-facing slope ([53,101,102]; electronic supplementary material, appendix S1). In evolution canyon models, the microclimatic inter-slope differences determine the level of gene flow among slopes and produce adaptive divergence [103,104]. Although the microclimatic and biotic contrasts from our study area are not as divergent as those from evolution canyons described by Nevo [99] in Israel—in which the two slopes unfold xeric, savannoid and shade, forested ecosystems—they seem to be enough to allow a genetic and phenotypic divergence within the blue tit population, suggesting that small variation in evolutionary canyons is sufficient to generate divergent evolution. A similar example is found in populations of citril finches (*Carduelis* (before *Serinus*) *citrinella*) in the Pyrenees, where birds from the north-facing slope are genetically and morphologically distinct from birds from the south-facing slope, which is drier and sunnier and has a lower abundance of pines [35,88]. In our study system, the inter-slope variation in clutch size [53] and the selection favouring such different clutch sizes between the two slopes of the valley suggest that blue tits face distinct selective pressures in each slope during their reproductive period. An important factor constraining all stages of avian reproduction is food availability [105]. Caterpillars are the main food source for blue tits during the spring [106–108], and we found that their abundance is lower in the east-facing slope [101,102]. Thus, food availability could be one of the most probable factors of selection underlying the phenotypic divergence observed between the two blue tit subpopulations and may explain why the clutch size is lower in the east-facing slope than in the west-facing slope of the valley.

## 5. Conclusions

We found genetic and phenotypic divergence among subpopulations of blue tits at surprisingly small scales (inside a continuous woodland). The topography and microclimatic geographic heterogeneity of our study area (a Mediterranean woodland located in a valley with two slopes) create different selective pressures when blue tits reproduce. The east-facing slope of the valley has a drier environment, with less tree cover and food availability, while the west-facing slope of the same valley exhibits a more humid environment with more tree cover and diversity, as well as higher caterpillar abundance. Consequently, blue tits adjust their reproductive effort to the slope where they breed by showing different clutch sizes [53]. Here, we provide evidence that an adaptive process seems to underlie such between-slope phenotypic variation. Concretely, blue tits from the opposite slopes are subtly genetically differentiated into two subpopulations, and selection (or other processes promoting divergence) seems to favour different clutch sizes between these subpopulations. This evolutionary scenario may seem surprising given that all the forestry formations of the woodland that cover the studied valley are connected, thus forming a continuum of habitats, and given that blue tits have a high dispersal potential.

## Data Availability

Data are available as supplementary material [109].

## References

[1] Clobert J , Baguette M , Benton TG , Bullock JM (eds). 2012 Dispersal Ecology and evolution. Oxford, UK: Oxford University Press.

[2] Slatkin M . 1987 Gene flow and the geographic structure of natural populations. Science **236** , 787–792. (10.1126/science.3576198) 3576198

[3] Clegg SM , Phillimore AB . 2010 The influence of gene flow and drift on genetic and phenotypic divergence in two species of Zosterops in Vanuatu. Phil. Trans. R. Soc. Lond. B **365** , 1077–1092. (10.1098/rstb.2009.0281) 20194170 PMC2830230

[4] Hutchison DW , Templeton AR . 1999 Correlation of pairwise genetic and geographic distance measures: inferring the relative influences of gene flow and drift on the distribution of genetic variability. Evolution **53** , 1898–1914. (10.1111/j.1558-5646.1999.tb04571.x) 28565459

[5] Slatkin M . 1993 Isolation by distance in equilibrium and non-equilibrium populations. Evolution **47** , 264–279. (10.1111/j.1558-5646.1993.tb01215.x) 28568097

[6] Edelaar P , Bolnick DI . 2012 Non-random gene flow: an underappreciated force in evolution and ecology. Trends Ecol. Evol. (Amst.) **27** , 659–665. (10.1016/j.tree.2012.07.009) 22884295

[7] Coulon A , Guillot G , Cosson JF , Angibault JMA , Aulagnier S , Cargnelutti B , Galan M , Hewison AJM . 2006 Genetic structure is influenced by landscape features: empirical evidence from a roe deer population. Mol. Ecol. **15** , 1669–1679. (10.1111/j.1365-294X.2006.02861.x) 16629819

[8] Ferrer ES , García-Navas V , Bueno-Enciso J , Barrientos R , Serrano-Davies E , Cáliz-Campal C , Sanz JJ , Ortego J . 2016 The influence of landscape configuration and environment on population genetic structure in a sedentary passerine: insights from loci located in different genomic regions. J. Evol. Biol. **29** , 205–219. (10.1111/jeb.12776) 26492434

[9] Quéméré E , Crouau-roy B , Rabarivola C , Louis Jr EE , Chikhi L . 2010 Landscape genetics of an endangered lemur (Propithecus tattersalli) within its entire fragmented range . Mol. Ecol. **19** , 1606–1621. (10.1111/j.1365-294X.2010.04581.x) 20345682

[10] Bolnick DI , Nosil P . 2007 Natural selection in populations subject to a migration load. Evolution **61** , 2229–2243. (10.1111/j.1558-5646.2007.00179.x) 17767592

[11] Lenormand T . 2002 Gene flow and the limits to natural selection. Trends Ecol. Evol. (Amst.) **17** , 183–189. (10.1016/S0169-5347(02)02497-7)

[12] Garant D , Forde SE , Hendry AP . 2007 The multifarious effects of dispersal and gene flow on contemporary adaptation. Funct. Ecol. **21** , 434–443. (10.1111/j.1365-2435.2006.01228.x)

[13] Nosil P , Vines TH , Funk DJ . 2005 Perspective: reproductive isolation caused by natural selection against immigrants from divergent habitats. Evolution **59** , 705–719. (10.1554/04-428) 15926683

[14] Postma E , van Noordwijk AJ . 2005 Gene flow maintains a large genetic difference in clutch size at a small spatial scale. Nature **433** , 65–68. (10.1038/nature03083) 15635410

[15] Blondel J , Dias PC , Perret P , Maistre M , Lambrechts MM . 1999 Selection-based biodiversity at a small spatial scale in a low-dispersing insular bird. Science **285** , 1399–1402. (10.1126/science.285.5432.1399) 10464098

[16] Coulon A , Fitzpatrick JW , Bowman R , Stith BM , Makarewich CA , Stenzler LM , Lovette IJ . 2008 Congruent population structure inferred from dispersal behaviour and intensive genetic surveys of the threatened Florida scrub-jay (Aphelocoma coerulescens). Mol. Ecol. **17** , 1685–1701. (10.1111/j.1365-294X.2008.03705.x) 18371014

[17] Garant D , Kruuk LEB , Wilkin TA , McCleery RH , Sheldon BC . 2005 Evolution driven by differential dispersal within a wild bird population. Nature **433** , 60–65. (10.1038/nature03051) 15635409

[18] García-Navas V , Ferrer ES , Sanz JJ , Ortego J . 2014 The role of immigration and local adaptation on fine-scale genotypic and phenotypic population divergence in a less mobile passerine. J. Evol. Biol. **27** , 1590–1603. (10.1111/jeb.12412) 24890737

[19] Porlier M , Garant D , Perret P , Charmantier A . 2012 Habitat-linked population genetic differentiation in the blue tit Cyanistes caeruleus. J. Hered. **103** , 781–791. (10.1093/jhered/ess064) 23087385

[20] Milá B , Wayne RK , Fitze P , Smith TB . 2009 Divergence with gene flow and fine-scale phylogeographical structure in the wedge-billed woodcreeper, Glyphorynchus spirurus, a Neotropical rainforest bird. Mol. Ecol. **18** , 2979–2995. (10.1111/j.1365-294X.2009.04251.x) 19538341

[21] Smith TB , Calsbeek R , Wayne RK , Holder KH , Pires D , Bardeleben C . 2005 Testing alternative mechanisms of evolutionary divergence in an African rain forest passerine bird. J. Evol. Biol. **18** , 257–268. (10.1111/j.1420-9101.2004.00825.x) 15715832

[22] Blondel J , Thomas DW , Charmantier A , Perret P , Bourgault P , Lambrechts MM . 2006 A thirty-year study of phenotypic and genetic variation of blue tits in Mediterranean habitat mosaics. Bioscience **56** , 661. (10.1641/0006-3568(2006)56[661:ATSOPA]2.0.CO;2)

[23] Dhondt AA , Adriaensen F , Matthysen E , Kempenaers B . 1990 Nonadaptive clutch sizes in tits. Nature **348** , 723–725. (10.1038/348723a0)

[24] Willi Y , Van Buskirk J , Hoffmann AA . 2006 Limits to the adaptive potential of small populations. Annu. Rev. Ecol. Evol. Syst. **37** , 433–458. (10.1146/annurev.ecolsys.37.091305.110145)

[25] Keller LF , Jeffery KJ , Arcese P , Beaumont MA , Hochachka WM , Smith JNM , Bruford MW . 2001 Immigration and the ephemerality of a natural population bottleneck: evidence from molecular markers. Proc. R. Soc. Lond. B **268** , 1387–1394. (10.1098/rspb.2001.1607) PMC1088753 11429139

[26] Szulkin M , Sheldon BC . 2008 Dispersal as a means of inbreeding avoidance in a wild bird population. Proc. R. Soc. B **275** , 703–711. (10.1098/rspb.2007.0989) PMC2596843 18211876

[27] Arnoux E , Eraud C , Navarro N , Tougard C , Thomas A , Cavallo F , Vetter N , Faivre B , Garnier S . 2014 Morphology and genetics reveal an intriguing pattern of differentiation at a very small geographic scale in a bird species, the forest thrush Turdus lherminieri. Heredity (Edinb.) **113** , 514–525. (10.1038/hdy.2014.56) 24984605 PMC4274614

[28] Björklund M , Ruiz I , Senar JC . 2010 Genetic differentiation in the urban habitat: the great tits (Parus major) of the parks of Barcelona city. Biol. J. Linn. Soc. **99** , 9–19. (10.1111/j.1095-8312.2009.01335.x)

[29] Menger J , Henle K , Magnusson WE , Soro A , Husemann M , Schlegel M . 2017 Genetic diversity and spatial structure of the rufous-throated antbird (Gymnopithys rufigula), an Amazonian obligate army-ant follower. Ecol. Evol. **7** , 2671–2684. (10.1002/ece3.2880) 28428858 PMC5395437

[30] Ortego J , García-Navas V , Ferrer ES , Sanz JJ . 2011 Genetic structure reflects natal dispersal movements at different spatial scales in the blue tit, Cyanistes caeruleus. Anim. Behav. **82** , 131–137. (10.1016/j.anbehav.2011.04.007)

[31] Camacho C , Canal D , Potti J . 2013 Nonrandom dispersal drives phenotypic divergence within a bird population. Ecol. Evol. **3** , 4841–4848. (10.1002/ece3.563) 24363908 PMC3867915

[32] Camacho C , Canal D , Potti J . 2016 Natal habitat imprinting counteracts the diversifying effects of phenotype-dependent dispersal in a spatially structured population. BMC Evol. Biol. **16** , 158. (10.1186/s12862-016-0724-y) 27503506 PMC4976508

[33] Dubuc-Messier G , Caro SP , Perrier C , van Oers K , Réale D , Charmantier A . 2018 Gene flow does not prevent personality and morphological differentiation between two blue tit populations. J. Evol. Biol. **31** , 1127–1137. (10.1111/jeb.13291) 29791058

[34] Recuerda M , Palacios M , Frías O , Hobson K , Nabholz B , Blanco G , Milá B . 2023 Adaptive phenotypic and genomic divergence in the common chaffinch (Fringilla coelebs) following niche expansion within a small oceanic island. J. Evol. Biol. **36** , 1226–1241. (10.1111/jeb.14200) 37485603

[35] Senar JC , Borras A , Cabrera J , Cabrera T , Björklund M . 2006 Local differentiation in the presence of gene flow in the citril finch Serinus citrinella. Biol. Lett. **2** , 85–87. (10.1098/rsbl.2005.0412) 17148333 PMC1617188

[36] Blanquart F , Kaltz O , Nuismer SL , Gandon S . 2013 A practical guide to measuring local adaptation. Ecol. Lett. **16** , 1195–1205. (10.1111/ele.12150) 23848550

[37] Kawecki TJ , Ebert D . 2004 Conceptual issues in local adaptation. Ecol. Lett. **7** , 1225–1241. (10.1111/j.1461-0248.2004.00684.x)

[38] Wadgymar SM , DeMarche ML , Josephs EB , Sheth SN , Anderson JT . 2022 Local adaptation: causal agents of selection and adaptive trait divergence. Annu. Rev. Ecol. Evol. Syst. **53** , 87–111. (10.1146/annurev-ecolsys-012722-035231) 37790997 PMC10544833

[39] Brommer JE . 2011 Whither P_ST_? The approximation of Q_ST_ by P_ST_ in evolutionary and conservation biology. J. Evol. Biol. **24** , 1160–1168. (10.1111/j.1420-9101.2011.02268.x) 21457173

[40] Pujol B , Wilson AJ , Ross RIC , Pannell JR . 2008 Are Q_ST_–F_ST_ comparisons for natural populations meaningful? Mol. Ecol. **17** , 4782–4785. (10.1111/j.1365-294X.2008.03958.x) 19140971

[41] Saether SA , Fiske P , Kålås JA , Kuresoo A , Luigujõe L , Piertney SB , Sahlman T , Höglund J . 2007 Inferring local adaptation from Q_ST_–F_ST_ comparisons: neutral genetic and quantitative trait variation in European populations of great snipe. J. Evol. Biol. **20** , 1563–1576. (10.1111/j.1420-9101.2007.01328.x) 17584249

[42] Antoniazza S , Burri R , Fumagalli L , Goudet J , Roulin A . 2010 Local adaptation maintains clinal variation in melanin-based coloration of European barn owls (Tyto alba). Evolution **64** , 1944–1954. (10.1111/j.1558-5646.2010.00969.x) 20148951

[43] Edelaar P , Alonso D , Lagerveld S , Senar JC , Björklund M . 2012 Population differentiation and restricted gene flow in Spanish crossbills: not isolation-by-distance but isolation-by-ecology. J. Evol. Biol. **25** , 417–430. (10.1111/j.1420-9101.2011.02443.x) 22239460

[44] Holand AM , Jensen H , Tufto J , Moe R . 2011 Does selection or genetic drift explain geographic differentiation of morphological characters in house sparrows Passer domesticus? Genet. Res **93** , 367–379. (10.1017/S0016672311000267) 21859501

[45] Kekkonen J , Jensen H , Brommer JE . 2012 Morphometric differentiation across house sparrow Passer domesticus populations in Finland in comparison with the neutral expectation for divergence. Ibis (Lond. 1859) **154** , 846–857. (10.1111/j.1474-919X.2012.01252.x)

[46] Lehtonen PK *et al* . 2009 Geographic patterns of genetic differentiation and plumage colour variation are different in the pied flycatcher (Ficedula hypoleuca). Mol. Ecol. **18** , 4463–4476. (10.1111/j.1365-294X.2009.04364.x) 19796331

[47] Santure AW , Ewen JG , Sicard D , Roff DA , Møller AP . 2010 Population structure in the barn swallow, Hirundo rustica: a comparison between neutral DNA markers and quantitative traits. Biol. J. Linn. Soc. **99** , 306–314. (10.1111/j.1095-8312.2009.01366.x)

[48] Edelaar P , Burraco P , Gomez-Mestre I . 2011 Comparisons between Q_ST_ and F_ST_—how wrong have we been? Mol. Ecol. **20** , 4830–4839. (10.1111/j.1365-294X.2011.05333.x) 22060729

[49] Foerster K , Valcu M , Johnsen A , Kempenaers B . 2006 A spatial genetic structure and effects of relatedness on mate choice in a wild bird population. Mol. Ecol. **15** , 4555–4567. (10.1111/j.1365-294X.2006.03091.x) 17107482

[50] Matthysen E , Van de Casteele T , Adriaensen F . 2005 Do sibling tits (Parus major, P. caeruleus) disperse over similar distances and in similar directions? Oecologia **143** , 301–307. (10.1007/s00442-004-1760-7) 15578228

[51] Parejo D , White J , Clobert J , Dreiss A , Danchin E . 2007 Blue tits use fledgling quantity and quality as public information in breeding site choice. Ecology **88** , 2373–2382. (10.1890/06-2000.1) 17918414

[52] Garroway CJ , Radersma R , Sepil I , Santure AW , De Cauwer I , Slate J , Sheldon BC . 2013 Fine-scale genetic structure in a wild bird population: the role of limited dispersal and environmentally based selection as causal factors. Evolution. **67** , 3488–3500. (10.1111/evo.12121) 24299402

[53] Garrido-Bautista J , Hernández-Ruiz C , Ros-Santaella JL , Pintus E , Bernardo N , Comas M , Moreno-Rueda G . 2023 Habitat-dependent breeding biology of the blue tit (Cyanistes caeruleus) across a continuous and heterogeneous Mediterranean woodland. Avian Research **14** , 100109. (10.1016/j.avrs.2023.100109)

[54] Excoffier L , Lischer HEL . 2010 Arlequin suite ver 3.5: a new series of programs to perform population genetics analyses under Linux and Windows. Mol. Ecol. Resour. **10** , 564–567. (10.1111/j.1755-0998.2010.02847.x) 21565059

[55] Falush D , Stephens M , Pritchard JK . 2007 Inference of population structure using multilocus genotype data: dominant markers and null alleles. Mol. Ecol. Notes **7** , 574–578. (10.1111/j.1471-8286.2007.01758.x) 18784791 PMC1974779

[56] Hubisz MJ , Falush D , Stephens M , Pritchard JK . 2009 Inferring weak population structure with the assistance of sample group information. Mol. Ecol. Resour. **9** , 1322–1332. (10.1111/j.1755-0998.2009.02591.x) 21564903 PMC3518025

[57] Pritchard JK , Stephens M , Donnelly P . 2000 Inference of population structure using multilocus genotype data. Genetics **155** , 945–959. (10.1093/genetics/155.2.945) 10835412 PMC1461096

[58] Evanno G , Regnaut S , Goudet J . 2005 Detecting the number of clusters of individuals using the software STRUCTURE: a simulation study. Mol. Ecol. **14** , 2611–2620. (10.1111/j.1365-294X.2005.02553.x) 15969739

[59] Earl DA , vonHoldt BM . 2012 STRUCTURE HARVESTER: a website and program for visualizing STRUCTURE output and implementing the Evanno method. Conserv. Genet. Resour. **4** , 359–361. (10.1007/s12686-011-9548-7)

[60] Kopelman NM , Mayzel J , Jakobsson M , Rosenberg NA , Mayrose I . 2015 CLUMPAK: a program for identifying clustering modes and packaging population structure inferences across K. Mol. Ecol. Resour. **15** , 1179–1191. (10.1111/1755-0998.12387) 25684545 PMC4534335

[61] Excoffier L , Smouse PE , Quattro JM . 1992 Analysis of molecular variance inferred from metric distances among DNA haplotypes: application to human mitochondrial DNA restriction data. Genetics **131** , 479–491. (10.1093/genetics/131.2.479) 1644282 PMC1205020

[62] Spitze K . 1993 Population structure in Daphnia obtusa: quantitative genetic and allozymic variation. Genetics **135** , 367–374. (10.1093/genetics/135.2.367) 8244001 PMC1205642

[63] Raeymaekers JAM , Houdt JKJ , Larmuseau MHD , Geldof S , Volckaert FAM . 2006 Divergent selection as revealed by P_ST_ and QTL-based F_ST_ in three-spined stickleback (Gasterosteus aculeatus) populations along a coastal-inland gradient. Mol. Ecol **16** , 891–905. (10.1111/j.1365-294X.2006.03190.x) 17284219

[64] McKay JK , Latta RG . 2002 Adaptive population divergence: markers, QTL and traits. Trends Ecol. Evol. (Amst.) **17** , 285–291. (10.1016/S0169-5347(02)02478-3)

[65] Merilä J , Crnokrak P . 2001 Comparison of genetic differentiation at marker loci and quantitative traits. J. Evol. Biol. **14** , 892–903. (10.1046/j.1420-9101.2001.00348.x)

[66] Botero‐Delgadillo E *et al* . 2017 Variation in fine‐scale genetic structure and local dispersal patterns between peripheral populations of a South American passerine bird. Ecol. Evol. **7** , 8363–8378. (10.1002/ece3.3342) 29075455 PMC5648682

[67] Li J , Lv L , Wang P , Wang Y , Hatchwell BJ , Zhang Z . 2019 Sex-biased dispersal patterns of a social passerine: complementary approaches and evidence for a role of spatial scale. Biol. J. Linn. Soc. **128** , 592–602. (10.1093/biolinnean/blz122)

[68] Ortego J , Aparicio JM , Cordero PJ , Calabuig G . 2008 Individual genetic diversity correlates with the size and spatial isolation of natal colonies in a bird metapopulation. Proc. R. Soc. B **275** , 2039–2047. (10.1098/rspb.2008.0475) PMC2596367 18505717

[69] Alcaide M , Serrano D , Tella JL , Negro JJ . 2009 Strong philopatry derived from capture-recapture records does not lead to fine-scale genetic differentiation in lesser kestrels. J. Anim. Ecol. **78** , 468–475. (10.1111/j.1365-2656.2008.01493.x) 19054221

[70] Adams RV , Burg TM . 2015 Influence of ecological and geological features on rangewide patterns of genetic structure in a widespread passerine. Heredity (Edinb.) **114** , 143–154. (10.1038/hdy.2014.64) 25074576 PMC4815624

[71] Adams RV , Lazerte SE , Otter KA , Burg TM . 2016 Influence of landscape features on the microgeographic genetic structure of a resident songbird. Heredity (Edinb.) **117** , 63–72. (10.1038/hdy.2016.12) 26905462 PMC4949724

[72] Lemoine M *et al* . 2016 Low but contrasting neutral genetic differentiation shaped by winter temperature in European great tits. Biol. J. Linn. Soc **118** , 668–685. (10.1111/bij.12745)

[73] Perrier C , Rougemont Q , Charmantier A . 2020 Demographic history and genomics of local adaptation in blue tit populations. Evol. Appl. **13** , 1145–1165. (10.1111/eva.13035) 32684952 PMC7359843

[74] de Greef E , Brashear W , Delmore KE , Fraser KC . 2022 Population structure, patterns of natal dispersal and demographic history in a declining aerial insectivore, the purple martin Progne subis. J. Avian Biol. **2022** , e02929. (10.1111/jav.02929)

[75] Edelaar P , Siepielski AM , Clobert J . 2008 Matching habitat choice causes directed gene flow: a neglected dimension in evolution and ecology. Evolution **62** , 2462–2472. (10.1111/j.1558-5646.2008.00459.x) 18637835

[76] Szulkin M , Zelazowski P , Nicholson G , Sheldon BC . 2009 Inbreeding avoidance under different null models of random mating in the great tit. J. Anim. Ecol. **78** , 778–788. (10.1111/j.1365-2656.2009.01544.x) 19383076

[77] Joly E . 2011 The existence of species rests on a metastable equilibrium between inbreeding and outbreeding: an essay on the close relationship between speciation, inbreeding and recessive mutations. Biol. Direct **6** , 62. (10.1186/1745-6150-6-62) 22152499 PMC3275546

[78] Ferrer ES , García-Navas V , Bueno-Enciso J , Sanz JJ , Ortego J . 2015 Multiple sexual ornaments signal heterozygosity in male blue tits. Biol. J. Linn. Soc. Lond. **115** , 362–375. (10.1111/bij.12513)

[79] Verheyen GR , Matthysen E , Adriaensen F , Broeckhoven C , Dhondt AA . 1997 Differentiation among blue tit (Parus caeruleus) populations measured with five minisatellite single locus probes. Belg. J. Zool. **127** , 179–189.

[80] Szulkin M , Gagnaire PA , Bierne N , Charmantier A . 2016 Population genomic footprints of fine-scale differentiation between habitats in Mediterranean blue tits. Mol. Ecol. **25** , 542–558. (10.1111/mec.13486) 26800038

[81] Senar JC , Björklund M . 2021 Recent spread of blue tits into the Barcelona urban environment: morphological differences and the role of balanced dispersal. Evol. Ecol. **35** , 83–99. (10.1007/s10682-020-10087-5)

[82] Ferrer ES , García-Navas V , Sanz JJ , Ortego J . 2016 The strength of the association between heterozygosity and probability of interannual local recruitment increases with environmental harshness in blue tits. Ecol. Evol. **6** , 8857–8869. (10.1002/ece3.2591) 28035274 PMC5192745

[83] Markowski M , Minias P , Bańbura M , Glądalski M , Kaliński A , Skwarska J , Wawrzyniak J , Zieliński P , Bańbura J . 2021 Genetic structure of urban and non-urban populations differs between two common parid species. Sci. Rep. **11** , 10428. (10.1038/s41598-021-89847-4) 34001959 PMC8128859

[84] Blondel J , Perret P , Dias PC , Lambrechts MM . 2001 Is phenotypic variation of blue tits (Parus caeruleus L.) in Mediterranean mainland and insular landscapes adaptive? Genet. Sel. Evol. **33** , S121–S139. (10.1186/BF03500877)

[85] Postma E , Den Tex RJ , Van Noordwijk AJ , Mateman AC . 2009 Neutral markers mirror small-scale quantitative genetic differentiation in an avian island population. Biol. J. Linn. Soc. **97** , 867–875. (10.1111/j.1095-8312.2009.01252.x)

[86] García-Navas V , Cáliz-Campal C , Ferrer ES , Sanz JJ , Ortego J . 2014 Heterozygosity at a single locus explains a large proportion of variation in two fitness-related traits in great tits: a general or a local effect? J. Evol. Biol. **27** , 2807–2819. (10.1111/jeb.12539) 25370831

[87] Van Bers NEM *et al* . 2012 The design and cross‐population application of a genome‐wide SNP chip for the great tit Parus major . Mol. Ecol. Resour. **12** , 753–770. (10.1111/j.1755-0998.2012.03141.x) 22487530

[88] Senar JC , Conroy MJ , Borras A . 2002 Asymmetric exchange between populations differing in habitat quality: a metapopulation study on the citril finch. J. Appl. Stat. **29** , 425–441. (10.1080/02664760120108791)

[89] Butlin RK *et al* . 2014 Parallel evolution of local adaptation and reproductive isolation in the face of gene flow. Evolution **68** , 935–949. (10.1111/evo.12329) 24299519 PMC4261988

[90] Clark JD , Benham PM , Maldonado JE , Luther DA , Lim HC . 2022 Maintenance of local adaptation despite gene flow in a coastal songbird. Evolution **76** , 1481–1494. (10.1111/evo.14538) 35700208 PMC9545442

[91] Yeaman S , Otto SP . 2011 Establishment and maintenance of adaptive genetic divergence under migration, selection, and drift. Evolution **65** , 2123–2129. (10.1111/j.1558-5646.2011.01277.x) 21729066

[92] Ferrer ES , García-Navas V , Sanz JJ , Ortego J . 2014 Individual genetic diversity and probability of infection by avian malaria parasites in blue tits (Cyanistes caeruleus). J. Evol. Biol. **27** , 2468–2482. (10.1111/jeb.12489) 25264126

[93] García-Navas V , Ortego J , Sanz JJ . 2009 Heterozygosity-based assortative mating in blue tits (Cyanistes caeruleus): implications for the evolution of mate choice. Proc. Biol. Sci. **276** , 2931–2940. (10.1098/rspb.2009.0417) 19474042 PMC2817209

[94] Olano-Marin J , Mueller JC , Kempenaers B . 2011 Correlations between heterozygosity and reproductive success in the blue tit (Cyanistes caeruleus): an analysis of inbreeding and single locus effects. Evolution **65** , 3175–3194. (10.1111/j.1558-5646.2011.01369.x) 22023584

[95] Sheldon BC , Kruuk LEB , Merilä J . 2003 Natural selection and inheritance of breeding time and clutch size in the collared flycatcher. Evolution **57** , 406–420. (10.1111/j.0014-3820.2003.tb00274.x) 12683536

[96] Van Der Jeugd HP , McCleery R . 2002 Effects of spatial autocorrelation, natal philopatry and phenotypic plasticity on the heritability of laying date. J. Evol. Biol. **15** , 380–387. (10.1046/j.1420-9101.2002.00411.x)

[97] Postma E , Visser J , Van Noordwijk AJ . 2007 Strong artificial selection in the wild results in predicted small evolutionary change. J. Evol. Biol. **20** , 1823–1832. (10.1111/j.1420-9101.2007.01379.x) 17714300

[98] Charmantier A , Doutrelant C , Dubuc-Messier G , Fargevieille A , Szulkin M . 2016 Mediterranean blue tits as a case study of local adaptation. Evol. Appl. **9** , 135–152. (10.1111/eva.12282) 27087844 PMC4780380

[99] Nevo E . 2006 “Evolution Canyon”: a microcosm of life’s evolution focusing on adaptation and speciation. Israel J. Ecol. Evol. **52** , 501–506. (10.1560/IJEE_52_3-4_485)

[100] Nevo E . 2009 Evolution in action across life at “Evolution Canyons”, Israel. Trends Evol. Biol. **1** , 3. (10.4081/eb.2009.e3)

[101] Garrido-Bautista J *et al* . 2022 Within-brood body size and immunological differences in blue tit (Cyanistes caeruleus) nestlings relative to ectoparasitism. Avian Res. **13** , 100038. (10.1016/j.avrs.2022.100038)

[102] Garrido-Bautista J , Soria A , Trenzado CE , Pérez-Jiménez A , Ros-Santaella JL , Pintus E , Bernardo N , Comas M , Moreno-Rueda G . 2021 Oxidative status of blue tit nestlings varies with habitat and nestling size. Comp. Biochem. Physiol. A Mol. Integr. Physiol. **258** , 110986. (10.1016/j.cbpa.2021.110986) 34023537

[103] Nevo E . 2012 “Evolution Canyon,” a potential microscale monitor of global warming across life. Proc. Natl Acad. Sci. USA **109** , 2960–2965. (10.1073/pnas.1120633109) 22308456 PMC3286920

[104] Nevo E . 2021 Evolution canyons model: biodiversity, adaptation, and incipient sympatric ecological speciation across life: a revisit. In New horizons in evolution (eds SP Wasser , MF Morgenstern ), pp. 291–348. London, UK: Elsevier Academic Press.

[105] Martin TE . 1987 Food as a limit on breeding birds: a life-history perspective. Annu. Rev. Ecol. Syst **18** , 453–487. (10.1146/annurev.es.18.110187.002321)

[106] García-Navas V , Ferrer ES , Serrano-Davies E . 2014 Experimental evidence for parental, but not parentally biased, favouritism in relation to offspring size in blue tits Cyanistes caeruleus. Ibis (Lond. 1859) **156** , 404–414. (10.1111/ibi.12140)

[107] Tremblay I , Thomas D , Blondel J , Perret P , Lambrechts MM . 2005 The effect of habitat quality on foraging patterns, provisioning rate and nestling growth in Corsican Blue TitsParus caeruleus. Ib. **147** , 17–24. (10.1111/j.1474-919x.2004.00312.x)

[108] Tremblay I , Thomas DW , Lambrechts MM , Blondel J , Perret P . 2003 Variation in blue tit breeding performance across gradients in habitat richness. Ecology **84** , 3033–3043. (10.1890/02-0663)

[109] Garrido-Bautista J , Comas M , Jowers MJ , Smith S , Penn DJ , Bakkali M *et al* . 2024 Supplementary material from: Fine-scale genetic structure and Phenotypic divergence of a Passerine bird population inhabiting a continuous Mediterranean woodland. Figshare (10.6084/m9.figshare.c.7262685) PMC11382889 39253402

